# Ether Spray in Strangulated Hernia

**Published:** 1866-12

**Authors:** 


### Ether Spray in Strangulated Hernia.

Dr. John Barclay reports in the *British Medical Journal*, a case of strangulated hernia, in which reduction was accomplished after the use of ether spray. The pain induced by the most gentle handling of the hernial tumor was so intense, that Dr. B. had to desist from taxis. Having brought with him Richardson's ether spray apparatus, thinking it might be useful in lieu of ice—it was determined to invert the patient, apply the ether spray short of freezing the skin, then to attempt the reduction, and, if failure was the result, to operate by the knife.

The head and shoulders then being supported on the floor by some pillows, and the buttocks raised as much as possible against an inclined plane, extemporized by an inverted bed-room chair, the ether spray was directed in the usual way on the swelling, for about forty seconds, when a minute spot of skin appeared white. The spray was at once removed, and on applying the fingers of the left hand on the swelling for about two seconds, accompanied by the most trifling pressure, plump up (or rather down) went the hernia, to the great delight and satisfaction of all. The man made a first rate recovery.—*Surgical Reporter.*

